# Annual Dinner of the New York Odontological Society

**Published:** 1885-03

**Authors:** 


### ANNUAL DINNER OF THE NEW YORK ODONTOLOGICAL SOCIETY.

*To the Editor of the Independent Practitioner:*

I had the good fortune to be a guest at the recent banquet of the Odontological Society, and should like to give to your readers some account of that gathering, and the impressions made upon me, besides acknowledging the debt of gratitude I owe to my entertainers. This society enjoys the enviable reputation of being one of the most influential dental societies in the world. Certainly it is the best advertised society on the planet. Its monthly proceedings are sought for with avidity, and are translated into all languages that have a dental literature. And this pre-eminence is richly deserved. Not a few of us remember how, in the past, a single session of the Odontological Society was worth more in its contributions to science than any session of any National Association that was ever held. The "New Departure," long in embryo in the minds and practice of individuals, was born into notice and life in the Odontological Society.

Whatever the Odontological Society does, it does royally. Its annual dinners have become a feature unequaled and unapproachable.

Well might it appear a royal banquet to a few travel-jaded souls who had spent four days and nights making a journey of a thousand miles in getting there; encountering blizzards from the Arctic circle, and snow-drifts well-nigh impassable.

It was on the night of the 18th of February that the society and its guests, to the number of seventy-five, assembled at Martinellis, a famous restaurant in New York, and greeted each other with all the cordiality of a fraternal family. From all over the land came eminent representatives of our profession to do honor to the occasion.

In this respect, and in its intellectual status, it was the most select gathering of dentists that it has been our fortune ever to behold.

The occasion was also honored by gentlemen representing other liberal professions, whose reputation is world-wide, and to look upon whose faces and listen to whose eloquence was alone worth the entire journey.

The perfection of the arrangements and the complete success with which they were carried out, is worthy of all praise. From beginning to end everything moved with military precision. The credit of this is largely due to that Napoleon of organizers and managers, Dr. Kingsley, who was facetiously dubbed "the Autocrat of the Odontological dinner," but who, as Chairman of the Committee of Arrangements, was ably supported by Dr. Lord and Dr. Hill.

At seven o'clock the guests were seated, and at half past nine it was evident that all possible animosities which might ever have existed had now been healed, and the fragrant order of the "cigar of peace" ascended with the curling clouds of smoke, and good fellowship and hilarity reigned. The lions are always docile when they have just been fully fed.

Dr. Jarvie, President of the Society, sat at the head of the table and conducted the exercises in an able and dignified manner.

With a brief welcome to the guests who had honored the occasion by their presence, he called upon the Chairman of the Committee of Arrangements, Dr. Kingsley, for a report. This report proved to be one of the features of the evening. For the ensuing ten minutes the audience were kept in a constant uproar of laughter by its humor, which embraced extracts from letters of regret, and telegrams of the most witty character.

The President then gave the toast of the evening: "Dentistry; a science born of medicine and the arts, which more than any other profession is indebted to *all* the sciences and *all* the arts for its best expression and attainments."

We were particularly struck with this sentiment, as being the most comprehensive statement of the. scientific status of dentistry that could have been made.

Dr. J. Smith Dodge, Jr., was called upon to respond. This gentleman is credited with being one of the most eloquent and accomplished orators in the dental profession, and certainly, if he had never earned that distinction before, his efforts at this gathering showed that he was fully entitled to it. Any attempt to epitomize his speech would only spoil it; it was a masterly effort of a half hour's length, which will amply repay publication in full, in your journal, if a copy can be obtained.

The second regular speaker was Dr. D. B. St. John Roosa, who, the President said, had made more deaf to hear and blind to see than any other man in New York.

Dr. Roosa spoke for the "Mother of Dentestry," and humorously said that it was the first time in his life that he had ever felt on an equality with the dentist. Heretofore, when in their presence, he had always been strapped and damned by their various apparatus, until he was powerless to resist; but now he could punish them by wagging his own jaw and boring them. He urged a benevolent and philanthropic spirit among dentists, which should show itself in the establishment of a dental infirmary for poor people, in every ward of the city.

Mr. F. N. Bangs spoke to the toast of the evening from the lawyers' standpoint, introducing much humor and fully sustaining his reputation of being one of the wittiest men at the bar.

One of the happiest hits of the evening was when Mr. Whitelaw Reid, of the *Tribune*, speaking for the press, said that there was a class of dentists that he did not see represented, one alone of which was of a great deal more value to the press than the entire body then present. He would not for the life of him dare enter *their* office, but he was most happy to meet them in his. Richmond had paid him five hundred dollars for a single advertisement, while not one of those before him had ever paid him one cent, and he didn't suppose they ever would.

The President introduced Mr. George Fawcett Rowe, as a fitting representative of all the arts, whose home was the world—journalist, correspondent, dramatist, actor, artist, etc., etc.

Mr. Rowe gave an amusing account of his observation of the teeth of the mummies in Egypt. From an inspection of several thousand of them (the exact number he did not remember—two or three more or less would not make much difference), and finding in the mouth of each thirty-two *sound* teeth, he concluded they did not have dentists in those days. If there had been dentists the results would probably have been different.

Dr. John B. Rich, one of the veteran dentists, but who looks in the very prime of his manhood, gave interesting reminiscences of dentistry as it was when he began practice, forty-five years ago, and contrasted the social standing of dentistry at that period with the present.

Following Dr. Rich came the genial Garretson, from the Quaker city, and unmistakably of Quaker stock. There never was a better exemplification of the fact that religion is largely a matter of temperament. The old ascetic Puritan could not be other than a Calvinist, and if Dr. Garretson could have been born of Calvinistic stock, he would certainly have gravitated into the peace-loving fold of the Quakers. There is no antagonism in an assembly when Dr. Garretson is speaking. His voice, manner, and words have all the charm of a delightful perfume, and the effect is that of oil on troubled waters. It is a rare advantage that a young man possesses to be under Dr. Garretson's influence. His remarks at the banquet showed that no dentist of education and culture need be at all anxious about his social standing. It is the man who elevates the occupation, not the occupation the man.

Dr. Barrett, whose commanding figure attracts attention in any assemblage, not more than his readiness and fluency of speech, followed Dr. Garretson, using for his theme, "Dental Literature."

No entertainment of dentists where Dr. Atkinson is would be a complete success without the sound of his voice, and this brief record of that banquet would be incomplete without a reference to him. Dr. Atkinson possesses a mind and qualities the counterpart of which cannot be found in the entire dental profession. His earnestness, his conscientiousness (as viewed from his own standpoint), his self-abnegation, his intense enthusiasm, his facility of speech, his profusion of ideas, his wondrous command of words, and the extravagance of his hyperbole, are unparalleled. To those who hear him but occasionally, his speeches are an entertainment never to be forgotten. On this occasion he made one of the best efforts of his life, thus adding to his already accumulated honors, and doing equal credit to the Odontological Society.

Other brief speeches were made by Drs. Allport, Shepard, etc., which time and space forbid any reference to, as this account is already so long that I fear you cannot find room for it.

One thing only occurred to mar the complete enjoyment of the festivities. Just before the close, the announcement was made of the sudden and possibly alarming illness of Dr. Dwinelle, who had been expected to speak of Dentistry as a scientific fine art.

A universal voice of sympathy was extended to him, and an involuntary prayer for his speedy recovery. PILGRIM.

